# Supplementary material for: Wound-Microenvironment Engineering through Advanced-Dressing Bioprinting
Source: Int J Mol Sci. 2022 Mar 4;23(5):2836. doi: 10.3390/ijms23052836 (PMC8911091; doi:10.3390/ijms23052836)
Supplement: Supplementary file 1 [file ijms-23-02836-s001.zip › ijms-1611310-supplementary.pdf]

Supplementary Material

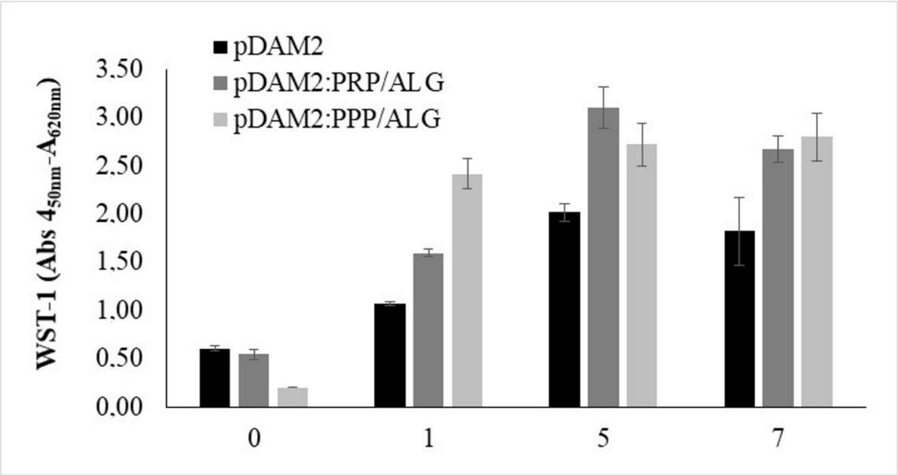

Figure S1. HDF metabolic activity encapsulated in pDAM2, pDAM2: PRP/ALG and pDAM2: PPP/ALG hydrogels at 0, 1, 5 and 7 days of culture and analyzed by WST-1 colorimetric assay.

Table S1. Primers for RT-PCR.

Gene (RNA)	Sequence	ID (Bio-Rad, Laboratories, Spain)
GAPDH (1.25 ng)	TCATTTCCTGGTATGACAACGAATTTGGCTACAGCAACAGGGTGGTGGAC-CTCATGGCCCACATGGCCTCCAAGGAGTAAGACCCCTGGACCACCAGCCCCAGCAAGAGCACAAGAGGAAGAG	dHsaCPE5031597
COL1A1 (2.5 ng)	TTGGTCTCGTCACAGATCACGTCATCGCACAACACCTTGCCGTTGTCGCAGAC-GCAGATCCGGCAGGGCTCGGGTTTCCACACGTCTCGGTCATGGTACCTGAGGCCGTTCTGTACGCAGGT	dHsaCPE5034390
COL1A2 (2.5 ng)	AGAGATGAACTGAGGTCCTTGTTTTGTTTTGTTCATAATACAAAGGTGCTAATTAA-TAGTATTTCAGATACTTGAAGAATGTTGATGGTGCTAGAAGAATTTGAGAAGAAATACTCCTGTATT	dHsaCPE5026780
COL3A1 (2.5 ng)	GATGAGATTATGACTTCACTCAAGTCTGTTAATGGACAAATAGAAAGCCTCATTAG-TCCTGATGGTTCTCGTAAAAACCCCGCTAGAAACTGCAGAGACCTGAAATTCTGCCATCCTGAACT	dHsaCPE5040292
COL4A1 (25 ng)	TTGTGACATTAGCTGAGTCAGGCTTCATTATGTTCTTCTCATACAGACTTGG-CAGCGGCTGACGTGCGTGCGCAGCTCCCCTGCCTTCAAGGTGGACGGCGTAGGCTTCTTGAACATCTCGC	dHsaCPE5049054
COL4A2 (25 ng)	GTGGGGTTCCTCGCCCAGAGCGTCTTGGCGGGTGTGAAGAAGTTT-GATGTGCCGTGTGGAG-GAAGAGATTGCAGTGGGGGCTGCCAGTGCTACCCTGAGAAAGGTGGACGTGGTCAGCCTGG	dHsaCPE5036732
COL4A3 (25 ng)	CCTGGGTAGGGGAAGAGAAGGGGGCATGTGGTATCCTGGAGCATTGTGTATA-GAACTGGAT-TTTCAGACCTGCTGAGGACCGTAAGGCCTGATGGAACACAGAACTGAACTGAGGTTCATGG	dHsaCPE5040370
ELN (25 ng)	GGATTCGCTCTAGCATCCCTCCTCTCCTGGGTCCACTTGGCCGTCTCCTCCCCAC-CGATCGCTGTTCCCCACATCTGGGGCGCTTTTGGGTTGGAAAACCACCCCACACTGGGAATAGCCACC	dHsaCPE5192509
FN1 (1.25 ng)	TGCCTCTCACACTTCCACTCTCCTCGGCCGTTGCCTGTGCAGATGCACTGGAG-CAGGTTTCCTCGATTATCCTTCTTGCTCCAGGTGTCTCCAATTCTATAGGATGTCCTTGTGTCCTGATCG	dHsaCPE5035056

Table S2. RT-PCR protocol.

	T° (°C)	Time	Ramp Rate	Number of Cycles

Hold	25	3 min	2 °C/sec	1
RT	48	60 min	2 °C/sec	1
Enzyme activation	95	10 min	2 °C/sec	1
Denaturation	95	30 sec	2 °C/sec	40
Annealing/extension	55	2 min	2 °C/sec	
Enzyme deactivation	98	10 min	2 °C/sec	1
Hold	4	Infinite	2 °C/sec	1
